# Age-dependent female responses to a male ejaculate signal alter demographic opportunities for selection

**DOI:** 10.1098/rspb.2013.0428

**Published:** 2013-09-07

**Authors:** Claudia Fricke, Darrell Green, Walter E. Mills, Tracey Chapman

**Affiliations:** 1 School of Biological Sciences, University of East Anglia, Norwich Research Park, Norwich NR4 7TJ, UK; 2 Institute for Evolution and Biodiversity, Westfaelische Wilhelms-University, Huefferstrasse 1, Muenster 48 149, Germany

**Keywords:** sexual selection, sexual conflict, sex peptide, age-dependent selection, senescence, postmating responses

## Abstract

A central tenet of evolutionary explanations for ageing is that the strength of selection wanes with age. However, data on age-specific expression and benefits of sexually selected traits are lacking—particularly for traits subject to sexual conflict. We addressed this by using as a model the responses of *Drosophila melanogaster* females of different ages to receipt of sex peptide (SP), a seminal fluid protein transferred with sperm during mating. SP can mediate sexual conflict, benefitting males while causing fitness costs in females. Virgin and mated females of all ages showed significantly reduced receptivity in response to SP. However, only young virgin females also showed increased egg laying; hence, there was a narrow demographic window of maximal responses to SP. Males gained significant ‘per mating’ fitness benefits only when mating with young females. The pattern completely reversed in matings with older females, where SP transfer was costly. The overall benefits of SP transfer (hence opportunity for selection) therefore reversed with female age. The data reveal a new example of demographic variation in the strength of selection, with convergence and conflicts of interest between males and ageing females occurring over different facets of responses to a sexually antagonistic trait.

## 1. Introduction

Ageing is a fundamental biological process manifested as an ever-increasing risk of mortality and decline in reproductive performance with age [1]. Evolutionary theory recognizes that ageing can be explained through a decrease in the strength of natural selection with age. This decrease permits the accumulation of late-life acting deleterious mutations in the germline [2] and/or alleles with beneficial early-life but deleterious late-life effects (i.e. antagonistic pleiotropy [3,4]). In these scenarios, ageing is a side-effect of selection focused on traits that maximize fitness, though in kin-selected contexts direct selection on ageing *per se* is also possible [5].

Whatever the predominant route by which ageing occurs, reproductive schedules are necessarily tightly linked with ageing patterns. In line with theory, evolutionary shifts in the optimum age for reproduction (e.g. by manipulating the age at which reproduction occurs) lead to predicable, directional changes in lifespan [1,6,7]. It has recently been proposed, however, that the role of sexual selection in ageing has been overlooked (reviewed in [8]). Within this context, sexual conflict is expected to have a particularly significant role in the evolution of ageing rates, and upon sex differences in ageing in particular. Sexual conflict arises because each sex can maximize their fitness in a way that results in the expression of significant costs in the other [9]. Males may, for example, gain from mating at a higher frequency than females, and frequent matings often lead to decreased female lifespan and reproductive success [10,11]. Under this scenario, the evolution of adaptations in one sex leads to selection for cost-reducing counter adaptations in the other [9,12,13]. This ‘antagonistic coevolution’ is a widespread and potent force for driving evolutionary change [14]. Sexual conflict has the potential to cause sex differences in ageing because of the negative effects of one sex on the lifespan of the other. For example, it is predicted that male-derived reductions in female lifespan should, over evolutionary time, lead to increased rates of ageing in females because of the increased hazards (risk of death) for females as they grow older [15].

A key prediction is that sexual conflict can push either or both sexes off their optimum life history in terms of lifespan and ageing patterns [8,15]. There have been no full tests of this idea to date, but the existing evidence is supportive. For example, in a study using artificial selection in seed beetles (*Acanthoscelides obtectus*) for early- and late-age of reproduction, males affected the rate of ageing in females in accordance with male interests [16]. Studies on *Callosobruchus maculatus* beetles also showed that virgin females, but not males, from lines subjected to elevated sexual selection had a higher baseline mortality rate [17]. Breeding experiments in the black field cricket (*Teleogryllus commodus*) revealed differences in the relationship between longevity and reproductive effort in males versus females [18]. Supporting data also come from a study using Neriid flies in which each sex aged most rapidly in an environment in which the other sex was in the majority [19].

To understand the role of sexual selection and sexual conflict in the evolution of ageing, we require measures of how traits subjected to sexual selection, and particularly sexual conflict, alter as individuals age. Studies have so far been conducted in the context only of mate choice in sexual selection. Theory predicts that as reproductive performance declines, older females will become less choosy. The empirical data are generally supportive, for example, female mating preferences often diminish with age, for example, in the cockroach *Nauphoeta cinerea* [20], the house cricket *Acheta domesticus* [21] and the guppy *Poecilia reticulata* [22]. *Drosophila melanogaster* males also gain lower last male sperm precedence when mating with older females [23]. By contrast, theory predicts that older males should increase in attractiveness [24,25]. However, here the empirical data are not consistently supportive. For example, older fathers can be discriminated against (e.g. in the sand fly *Lutzomyia longipalpis* [26]) show reduced mating success (e.g. in *C. maculatus* seed beetles [27]) and reduced offspring viability (e.g. in *D. melanogaster* [28]). A resolution for these inconsistent findings may lie in the suggestion that older males could accumulate germline mutations, resulting in a negative correlation between somatic and germline mutation load. For example, recent theory predicts that if germline mutation load is high in old males, then females can evolve a preference for younger males [29].

There is little work so far, however, on ageing and adaptations that can be subject to sexual conflict. This is an important omission because, as seen above, selection on such traits may provide a novel explanation of sex-specific rates of ageing if benefits and costs alter significantly with age and show contrasting patterns for each sex. Potential variation in the expression of costs and benefits with age has been observed [30]. Young females derived fecundity benefits from, and were least susceptible to, the deleterious effects of remating. By contrast, older females (more than 40 days after eclosion) benefitted less from remating and appeared to suffer increased survival costs of mating [30]. These findings highlight the potential for sex-specific selection on ageing rates.

Here, we examined the responses of females to sex peptide (SP), a seminal fluid protein transferred by males during mating. Receipt of SP causes diverse changes to female behaviour and physiology. It decreases female sexual receptivity [31,32]; increases egg production [31,32], juvenile hormone levels [33], feeding rate [34], sperm retention in storage [35] and antimicrobial peptide production [36]; and alters feeding preferences [37] and water balance [38]. However, to date, these effects have typically been demonstrated in ‘one-shot’ tests using young individuals, and nothing is yet known about variation across the life history. SP is of interest in the context of sexual conflict because it benefits males [39] but its receipt can result in costs for females [40,41]. It is therefore likely to be a significant contributor to male-derived mating costs in females in general [42]. Specifically, we tested (i) whether females retained the capacity to respond to SP as they age, both as virgins and as mated individuals and (ii) whether the benefits to males of transferring SP varied significantly across matings with young, middle-aged and old females, measured in a relevant competitive context.

## 2. Methods

### (a) Fly stocks

#### (i) Wild-type flies

Dahomey wild-type was collected in the 1970s in Benin, Africa and has been maintained since then at 25°C on a 12 D : 12 L cycle in large cage cultures under a regime of overlapping generations. Stocks were cultured in glass bottles (189 ml each) containing 70 ml of standard sugar–yeast (SY) food (100 g autolysed yeast powder, 100 g sucrose, 20 g agar, 30 ml Nipagin (10% w/v solution), 3 ml propionic acid, 1 l water). All experiments were conducted at 25°C in a humidified constant temperature room, using glass vials (75 mm height × 25 mm diameter) containing 7 ml of SY food with ad libitum live yeast granules or paste. To collect experimental adults, eggs were collected from females ovipositing on agar–grape juice plates (50 g agar, 600 ml red grape juice, 42.5 ml Nipagin (10% w/v solution), 1.1 l water) containing a smear of yeast paste. First-instar larvae emerging from these eggs were then cultured at a density of 100 larvae per vial. Virgin adults were collected, sorted using ice-anaesthesia, and held in groups of 10 in single sex groups until use.

#### (ii) Sex peptide-lacking males

*SP* knockout lines [31] were produced by crossing *SP*^0^/*TM3,Sb,ry* males to *Δ130/TM3,Sb,ry* females. The resulting *SP*^0^/*Δ130* (*SP*^0^) males produce no SP [31]. Control males were generated by crossing *SP*^0^,*SP^+^/TM3,Sb,ry* males to *Δ130/TM3,Sb,ry* females to generate genetically matched SP-producing *SP*^0^,*SP^+^/Δ130* (*SP^+^*) control males. The strains were backcrossed into the Dahomey wild-type. The *Δ130/TM3,Sb,ry* stock was backcrossed for three generations, and chromosomes 1, 2 and 4 of the *SP*^0^/*TM3,Sb,ry* and *SP*^0^,*SP^+^/TM3,Sb,ry* stocks were backcrossed for four generations. To generate *SP*^0^ and *SP^+^* males for experiments, three each of parental males and females described above were placed together in vials and transferred onto fresh food every day. Ten days later, *SP*^0^ and *SP^+^* male offspring were collected and housed in groups of 10 in vials until use.

### (b) Effect of sex peptide transfer on virgin and mated female fecundity and receptivity with age

#### (i) Responses to sex peptide in virgin females with age

Dahomey females (*n* = 650) were collected as virgins and held in groups of 10 per vial. These females were transferred onto fresh food every other day until used in the experiments. Twice a week, we tested the effects of receipt of SP on female remating and egg laying, using separate, randomly selected, independent groups of females for each time point. Female responses to receipt of SP were tested at age 1, 5, 8, 12, 15, 19, 22, 26, 29, 33, 36 and 40 days after eclosion. For each test, 25 virgin females were placed with either two *SP^+^* or *SP*^0^ males. New males were collected each week and, thus, all females were tested with young males (less than 8 days post eclosion). The introduction times, beginning and end of each matings were recorded (*n* = 15–20 matings per treatment per age). The proportion of matings within the first hour of observation was scored. Among the mated pairs, the males were then removed, and females allowed to lay eggs. Nineteen hours after their first mating, females were given the opportunity to remate for 1 h with two Dahomey wild-type males each. The number of females remating and the start and end of each mating were scored. The number of eggs was also counted, and vials were incubated for 12 days to allow all offspring to develop. Vials were then frozen for later counting of offspring.

#### (ii) Responses to sex peptide in mated females with age

In a second set of experiments, we repeated the above mating assays, using non-virgin females (detailed methods in the electronic supplementary material). Females were mated to wild-type males either ‘early’ or ‘late’ in their lives (at 2 days of age or 2 days before the *SP^+^*/*SP*^0^ test matings, respectively). This allowed us to investigate whether virgin and mated females of different ages (5, 12, 19, 26, 33 days of age, *n* = 40 per treatment) responded similarly to SP and whether the age at which the first mating occurred had any effect.

### (c) Fitness of sex peptide-transferring males held under competitive conditions with young, middle-aged or old females

Finally, we tested SP-dependent male reproductive success under competitive conditions with females of different ages. Separate, randomly drawn and independent groups of virgin females were allowed to age for either 3 (young), 12 (middle-aged) or 28 (old) days. *SP*^0^ or *SP^+^* males were each paired with individual females of each age class together with a single competitor male. Competitor males carried a dominant *Stubble* mutation (*Sb*^1^ backcrossed into the Dahomey wild-type four times, resulting in approx. 94% of its genome being rendered wild-type) to assign offspring paternity. Stubble is a dominant, homozygous lethal mutation—therefore the *Sb*^1^ males used were heterozygotes. To estimate wild-type focal male paternity, we multiplied by 2 the number of *Stubble* offspring and subtracted this from the total number of offspring produced. The trios of one female, one *SP*^0^ or *SP^+^* male and one competitor male were allowed to interact freely and were transferred to new vials every day for one week. We incubated the vacated vials for 12 days, then froze the emerging offspring for subsequent scoring of offspring number and paternity. We also measured male mating frequency by performing spot-checks of behaviour every 20 min for 3 h after lights on every morning. To distinguish between the two males, we clipped the wing tips of the competitor males. Thus, we measured a focal *SP*^0^ or *SP^+^* male's pre- as well as postmating success in a relevant competitive environment, to provide a robust estimate of male reproductive success.

### (d) Statistical methods

Data were analysed using R v. 12.2 [43]. Generalized linear models were used with the appropriate error structure and correction for overdispersion if necessary. Female age, mating treatment and male genotype were fixed factors. We first analysed the full models and tested significance of factors and interactions by excluding in turn each term and comparing the full with the reduced models. The deviance (*G*^2^) for each term was tested for significance by comparison with a chi-squared or *F*-distribution (when using the quasi-extension for overdispersed data [44]). For the analysis of male reproductive success, we included replicates in which offspring were produced for at least 3 days. To calculate a ‘per mating’ reproductive success index for *SP*^0^ versus *SP^+^* males, we followed the methods outlined in Fricke *et al*. [39]. For this, we calculated the ratio between the mean relative number of offspring and the mean relative number of matings gained by *SP*^0^ or *SP^+^* males. Significance testing was then performed using bootstrap resampling, to test for differences in the ‘per mating’ reproductive success with females in the young and old female age classes. We used the raw data for offspring number and mating rate for *SP*^0^ or *SP^+^* males. We then used the ‘Poptool’ extension in Excel to recalculate the difference in per mating male reproductive success for *SP*^0^ versus *SP^+^* males in 10 000 iterations, separately for the young and old age classes of females. The significance test was obtained by determining how often we obtained a value equal to or smaller than the observed difference in per mating male reproductive success for *SP*^0^ and *SP^+^* males. Error propagation was used to produce standard error estimates for the single per mating averages. We used the formula *Δz/z* = sqrt((*Δx/x*)^2^ *+* (*Δy/y*)^2^), whereby *x* represents mean focal offspring gain, *y* mean total offspring production and *z* = *x/y*. *Δx,* *Δy* are the standard errors for each of the two variables, whereas *Δz* is the new calculated standard error. It should be noted, however, that these standard error estimates based on single averages are necessarily very conservative.

An additional approach taken to compare per mating reproductive success was to combine five replicates (or four if we had to exclude a replicate) for each treatment to calculate a ‘per mating’ reproductive success for these combined sets of replicates (*n* = 6 per treatment). These indices were then compared using a Scheirer–Ray–Hare test (a non-parametric equivalent of the two-way ANOVA). We added one to all mating rate data to remove zeros in cases where we had observed offspring from both males. Means and standard errors are presented unless otherwise stated. Standard errors for proportion data were calculated as square-root [p*(1 − *p*)/*n*], where *p* is the proportion of females remating and *n* the number of trials in that particular test.

## 3. Results

### (a) Responses to sex peptide in ageing virgin and mated females

#### (i) Effect of sex peptide transfer on fecundity and receptivity of ageing virgin females

**Figure 1.**
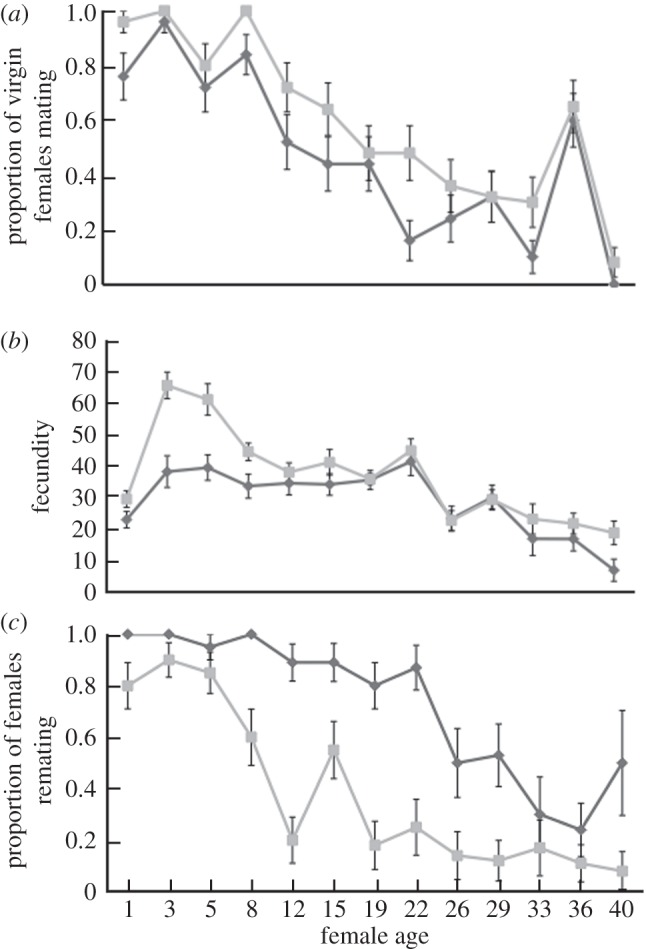
Responses of virgin females. (*a*) Number of virgin females (±s.e.m.) of increasing age mating with either SP-lacking (*SP*^0^, dark grey) or control males (*SP^+^*, light grey). Shown is the proportion of females mating within 1 h of introduction to males. (*b*) Fecundity (mean number of eggs ± s.e.m.) laid by females in the 19 h period following matings to either *SP*^0^ (dark grey) or *SP^+^* (light grey) males. (*c*) Proportion of females remating (±s.e.m.) with wild-type males within 1 h of introduction 19 h following matings to either *SP*^0^ (dark grey) or *SP^+^* (light grey) males.

There was, as expected, an overall general decline in the reproductive activity of virgin females as they aged (figure 1). Virgin females showed a significant decline in willingness to mate with age (female age: *G*^2^_12_ = 250.72, *p* < 0.0001; figure 1*a*), and there was also a significant age-related decline in fecundity (female age: *G*^2^_12_ = 1633.5, *F* = 18.18, *p* < 0.0001). Genetic backgrounds of the males were controlled; however, there were fewer matings at all ages between virgin females and *SP*^0^ in comparison with *SP*^+^ males (male genotype: *G*^2^_1_ = 15.45, *p* = 0.0001; interaction term: *G*^2^_12_ = 13.45, *p* = 0.337; figure 1*a*). Similarly, with increasing age mating latency became significantly longer, copulation durations became shorter and egg fertility (egg-to-adult survival) significantly lower (see the electronic supplementary material, figure S1*a–e*).

In terms of responses to SP, young virgin females showed significantly increased fecundity upon SP receipt, as expected. However, this effect diminished significantly with female age (figure 1*b*). This was evident as a significant effect of male genotype (GLIM dispersion parameter = 7.46, *G*^2^_1_ = 216.3 *F* = 28.88, *p* < 0.0001) and a marginally non-significant interaction between female age and male genotype (*G*^2^_12_ = 150.9, *F* = 1.69, *p* = 0.068). Consistent with the results for first matings, the proportion of females from both treatments remating significantly decreased with female age (female age: *G*^2^_12_ = 167.96, *p* < 0.0001). Within treatments, a significantly higher proportion of females remated following first matings with *SP*^0^ males (male genotype: *G*^2^_1_ = 83.22, *p* < 0.0001), as expected. However, there was no significant interaction of first male genotype with female age (interaction: *G*^2^_12_ = 11.69, *p* = 0.471; figure 1*c*). Hence, receipt of SP significantly reduced female receptivity to remating at all ages.

Overall, the results showed that SP transfer stimulated fecundity only in relatively young females (figure 1*b*), but significantly decreased receptivity to remating in females of all ages (figure 1*c*).

#### (ii) Effect of sex peptide transfer on fecundity and receptivity of ageing mated females

In contrast to the results for virgins described above, fecundity was insensitive to receipt of SP in both early- and late-mated females of all ages (see the electronic supplementary material, table S1 and figure S2*a*). Receipt of SP did, however, significantly reduce female remating receptivity at all female ages, as was again found in virgin females (see the electronic supplementary material, table S2 and figure S2*b*). SP receptivity responses were therefore independent of previous mating history (i.e. virgin or mated). The results from the tests with mated females therefore show that opportunities for males to significantly boost fecundity in non-virgin females via SP transfer were minimal, if any. By contrast, the period during which SP could act to significantly reduce female receptivity in mated females was unrestricted and independent of female age. As in virgins, non-virgin females also tended to exhibit an overall significant age-dependent decline in female fecundity and reproductive performance in general (see the electronic supplementary material, section S2*b*, table S3 and figure S3*a–f*).

### (b) Fitness of sex peptide-transferring males held under competitive conditions with young, middle-aged or old females

**Figure 2.**
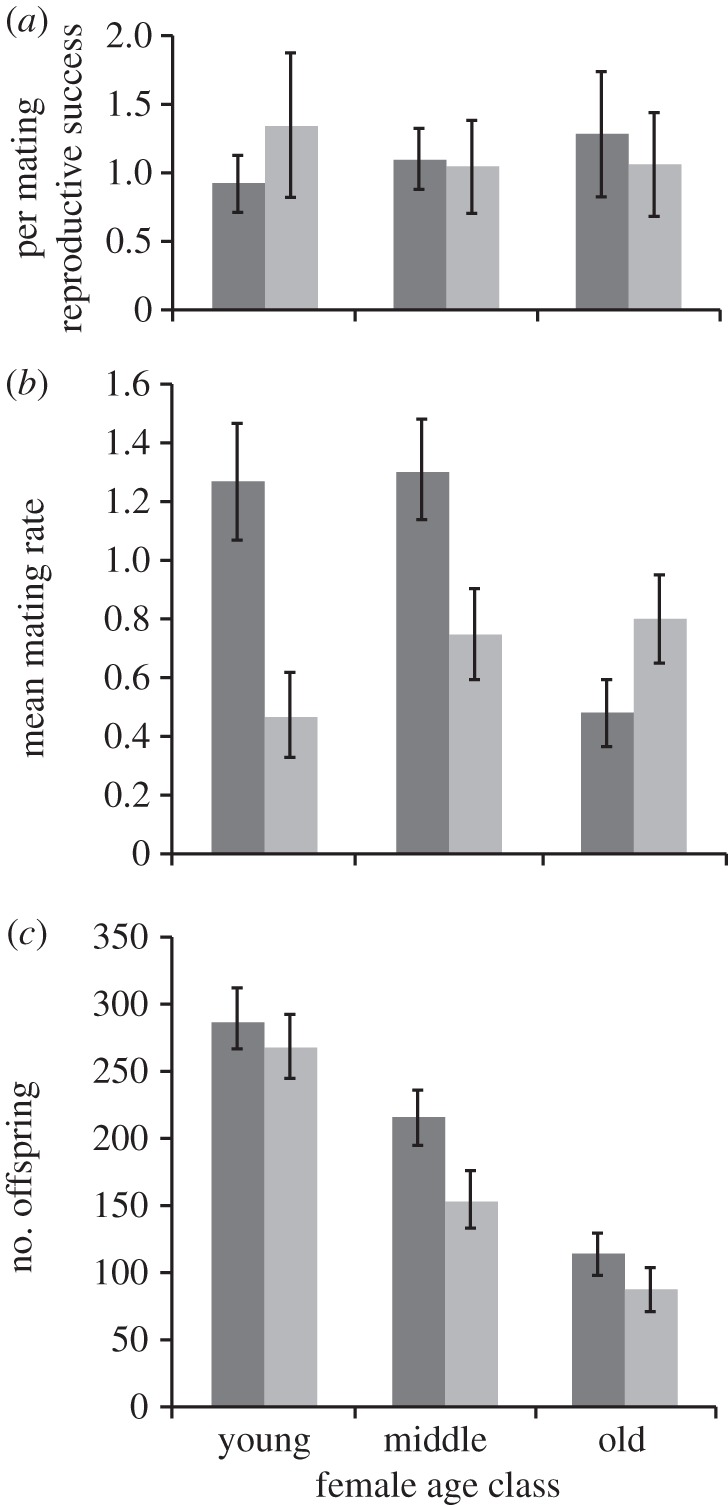
Reproductive success of SP-lacking (*SP*^0^, dark grey) and control (*SP^+^*, light grey) males each held together with independent groups of competitor males and young, middle-aged and old females. (*a*) Male ‘per mating’ reproductive success (mean ± s.e.m.) for *SP*^0^ (dark grey) and *SP^+^* (light grey) males held for a 6-day period with young (3 days), middle-aged (12 days) or old (28 days) females in a competitive context. For details of error bar calculations, see text. (*b*) The number (mean ± s.e.m.) of *SP*^0^ (dark grey) and *SP^+^* (light grey) matings observed during 3 h spot-checks every morning over the 6-day period of the male reproductive success assay. (*c*) The mean (±s.e.m.) number of offspring produced by *SP*^0^ (dark grey) or *SP^+^* (light grey) males over the entire 6-day assay period when held with either young, middle-aged or old females and a competitor male.

SP-transferring males achieved significantly higher ‘per mating’ reproductive success than SP-lacking males in matings with young females (figure 2*a*; bootstrap test, *p* = 0.0062). However, this effect levelled out in middle-aged and reversed in old females (note though that the bootstrap test for old females was not significant; *p* = 0.114). There was a marginally non-significant interaction between female age and male genotype (Scheirer–Ray–Hare test: *H* = 3.02; d.f. = 2, *p* = 0.082), which supports the trend for a reversal in the per-mating fecundity benefits of SP transfer in young versus old females. Overall, female age did not significantly affect male per mating reproductive success (female age: *H* = 1.36, d.f. = 2, *p* = 0.244; male genotype: *H* = 0.001, d.f. = 1, *p* = 0.976).

The same reversal across female age in the benefits of SP transfer was also seen for mating rate. There were significantly higher numbers of matings in the SP-lacking groups held with young and middle-aged females, but this pattern was reversed in groups containing old females. This effect was entirely driven by the *SP*^0^ and *SP^+^* males, independent of the ability of competitor males to transfer SP (see the electronic supplementary material, table S4 and figure 2*b*).

As before, female fecundity dropped significantly with age (female age: *G*^2^_2_ = 5212.5, *F* = 39.67, *p* < 0.0001; dispersion parameter = 66.40). SP-lacking males achieved significantly higher absolute reproductive success (total number of offspring produced: male genotype: *G*^2^_1_ = 299.95, *F* = 4.57, *p* = 0.034; interaction term: *G^2^_2_* = 120.78, *F* = 0.91, *p* = 0.405) driven by higher numbers of offspring gained by SP-lacking males held with middle-aged females (figure 2*c*). By contrast, the number of offspring fathered by the competitor males was mostly attributable to female age (*G*^2^_2_ = 3858.5, *F* = 21.81, *p* < 0.0001) and was not significantly affected by the genotype of the focal male (*G*^2^_1_ = 32.03, *F* = 0.36, *p* = 0.55). SP-lacking and control males did not differ significantly in the share of paternity they gained, nor was this affected by female age (all *p* > 0.14).

Overall, the results show, in tests conducted under realistic competitive conditions, reversals in the potential benefits of SP available to males as females age.

## 4. Discussion

The results show that female responses to receipt of SP changed significantly with age. However, the pattern was divergent for fecundity versus receptivity SP phenotypes. These age-related changes in female responsiveness to SP significantly altered the benefits males could gain from SP transfer. Males had only a narrow demographic window in which SP receptivity and egg responses were maximized and hence in which to gain significant benefits. In addition, there were significant costs of SP transfer to males in matings with older females that were relatively unresponsive to SP.

Virgin females exhibited mounting unresponsiveness to the fecundity-enhancing effects of SP with age, and became completely unresponsive beyond 8–10 days old (figure 1*b*). In already-mated females, this effect was even stronger, and SP receipt did not significantly enhance fecundity at all (see also [45]). This unresponsiveness could arise, because females became less sensitive to SP with increasing age. An alternative, non mutually exclusive explanation is that as fecundity decreases with advancing age, females lose the capacity to mount a response to receipt of SP—for example, if their reproductive tracts are already working at maximum capacity for that age. By contrast, the effect of SP transfer on remating receptivity was significant at all female ages and conditions tested. Hence, the two SP responses on which we focused—increased fecundity and decreased receptivity to remating [31,46]—became decoupled as females grew older. Uncoupling of SP phenotypes has previously been described in the context of nutritional manipulations—with fecundity enhancement, but not receptivity suppression, being strongly dependent on female nutritional state [41]. This uncoupling could result, as suggested above, from a loss in capacity to respond to some but not all facets of SP. Alternatively, uncoupling could be due to a divergence in male and female interests, as explored in more detail below.

Delays to female remating following SP transfer can benefit males by increasing their ‘per mating’ reproductive success [39] and delaying the onset of sperm competition. A significant reduction in the number of matings resulting from responses to SP receipt may also be beneficial for females. Each additional mating may contribute to mating costs in females [47], thus a delay in remating could be in the shared interest of both males and females. Although effective at all ages, SP transfer caused the smallest reduction in remating rate in young females. We suggest that the interests of males and females are more strongly aligned in middle-aged females, with the potential for conflict over mating decisions being higher in young females. For example, younger females are more fecund and as a consequence might be subject to increased harassment from males.

We considered the benefits for males transferring SP in matings with females from young, middle-aged and old female age classes in a relevant competitive environment. SP-transferring control males gained significant fitness benefits during a narrow window of only approximately 7 days when mating with young females. During this period, SP transfer significantly reduced mating rate. This, combined with fecundity responses, resulted in significantly higher fitness for SP-transferring males mating with young females. This period coincided with the strongest fecundity responses to SP, suggesting that egg-laying rate is an important fitness determinant in this context.

The significant decline in egg-laying rate reduced the potential fitness benefits for males of mating with older females, which is consistent with the declining strength of selection with age. SP-mediated behaviours or responses in individuals of approximately more than 30 days of age are therefore unlikely to be strongly selected due to the lack of potential benefits. Such effects can be estimated empirically by calculating fitness indices in which the Malthusian parameter, *r*, has decreasing weight with advancing age. The incorporation of such weights does not, however, alter the magnitude of difference between females mated to either *SP*^+^ or *SP*^0^ males, but instead emphasizes the diminishing contribution to fitness of individuals of ever-increasing age [1–4]. Such calculations can also be useful to consider the age-related fitness profiles of both sexes. Consistent with the idea that males are sensitive to female age-related fitness benefits, are findings such as the discrimination by males against matings with older females based on their cuticular hydrocarbon (CHC) profile [48], and ejaculate tailoring in which more sperm are transferred into young in comparison with old females [49]. It is possible therefore that in our experiments part of the decline in SP responses over time was actually due to males decreasing their investment in ejaculate in matings with older females [49]. Arguing against this, however, is that despite the dichotomy in SP ejaculate investment imposed by the treatments, fecundity converged in *SP^+^* and *SP*^0^ by about 12 days of age, whereas overall investment in fecundity started to rapidly decline only once females exceeded 30 days of age. There was no evidence therefore for graded, continual decreases in SP investment over time. The possibility of facultative investment according to variation in female age would nevertheless be interesting to investigate further in explicit tests.

SP also significantly reduced mating rate in middle-aged females in the competitive assay. However, there were no detectable per mating fitness benefits, presumably because of the lack of associated fecundity responses to SP. In absolute terms, SP-lacking males actually achieved higher reproductive success at this age. However, such potential benefits are likely to be offset by energetic demands in those males arising from increased courtship and mating.

This lack of fitness benefits for SP-transferring control males despite the induction of significantly reduced female remating rate across all ages illustrates that female age structure and corresponding fecundity patterns need to be taken into account when considering the effectiveness and fitness gains of a male sexual trait. The effectiveness with which refractoriness is induced may be diminished if it is combined with a general decline in willingness to mate in the first place. It is true that the fitness benefit to males arising from the reduction in female receptivity declined with the age of his partners. However, it should always benefit a male to prevent his partners from remating with other males, unless there are significant or rising costs of SP production for males with age and/or that the benefits of reducing female receptivity become zero because they are too old to engage in any further mating. The pertinent question then becomes whether there are there any benefits to be gained by not producing SP and whether males regulate the amount of SP transferred according to the age (strictly, likelihood of remating) of their partners. There are as yet no data to suggest this, but as noted above, further explicit tests of these ideas are needed.

In older females, SP transfer actually became costly for males. The tests with virgin and mated females suggest that this was associated with reduced fecundity responses to SP. Older females could be incapable of increasing fecundity significantly after receipt of SP, or may have evolved resistance to SP because of potentially greater costs [13,46,50]. In the competitive assay, unlike in the earlier tests, receptivity of older females was also apparently insensitive to SP receipt. Furthermore, older females receiving SP even exhibited significantly higher mating frequencies in the competitive tests. The results highlight the significant decline in female responses to SP with age, associated with increasing costs of SP transfer for males.

In terms of conflicts of interest between males and females over different facets of SP responses, we hypothesize that it might be in the interests of both sexes to shut off receptivity in response to SP receipt, but that there is less ‘agreement’ about investment patterns in fecundity (males will always benefit, whereas females might not). The uncoupling of SP traits might therefore be a manifestation of conflict. Males are apparently unable to boost fecundity once it has already been initiated (in mated females). However, they can still use SP to shut off receptivity, but with little apparent benefit, and increasing costs. Therefore, males do not gain the full potential benefits from SP transfer. Together, these findings suggest that there will be dynamic selection pressures acting on males depending on the demographic composition of the population.

Our study revealed clear evidence that female ageing alters the costs and benefits of a sexually antagonistic trait. Female ageing will therefore affect the opportunity and intensity of sexual conflict. It is also important to explore how this may affect ageing patterns *per se*. The sustained SP receptivity responses seen in middle-aged females could reduce mating-induced risks of mortality and thus decelerate the rate of senescence in females. Receipt of SP will also increase the amount of male harassment experienced by females, shortening their lives, while simultaneously selecting for increased lifespan in males owing to the longer average time between matings. Males, on the other hand, may be encumbered with a sexually antagonistic trait that has restricted effectiveness and that can carry significant costs including increased senescence [8,15]. There are therefore strong opportunities for sexually antagonistic coevolution to drive the evolution of sex-specific ageing rates, depending on the demographic make-up of a population and the interplay between SP transfer and SP responses in females of varying age.

## Data accessibility

All data for this study have been archived in the Dryad data repository (doi:10.5061/dryad.6sk71).
